# (5R*,11R*)-5-Methyl-1,2-dihydro-5,11-methano-5*H*,11*H*-1,3-thia­zolo[2,3-*d*][1,3,5]benzoxadiazo­cine

**DOI:** 10.1107/S1600536809044729

**Published:** 2009-10-31

**Authors:** Viktor Kettmann, Jan Světlík, Lucia Veizerová

**Affiliations:** a Faculty of Pharmacy, Comenius University, Odbojarov 10, SK-83232 Bratislava, Slovakia

## Abstract

The title compound, C_13_H_14_N_2_OS, crystallizes as a racemate in a non-chiral space group. It represents a conformationally restricted analogue of so-called Biginelli compounds known to exhibit multiple pharmacological activities and was selected for a single-crystal X-ray analysis in order to probe the chemical and spatial requirements of some kinds of activity. It was found that the state of hybridization of the formally aminic nitro­gen of the heterocycle is between *sp* ^2^ and *sp* ^3^ with the lone-pair electrons partially delocalized through conjugation with the sulfur atom rather than the double bond of the pyrimidine nucleus. As a result, the thia­zolo ring adopts a flat-envelope conformation and the puckering of the central pyrimidine ring is close to a half-chair. The critical phenyl ring is fixed in a pseudo-axial and perpendicular [dihedral angle 84.6 (1)°] orientation with respect to the pyrimidine ring *via* an oxygen bridge.

## Related literature

For typical bond lengths, see: Abrahams (1956 ▶); Burke-Laing & Laing (1976 ▶). For the pharmacological activity of Biginelli compounds, see: Deres *et al.* (2003 ▶); Kappe (2000 ▶). For the synthesis of rigid dihydro­pyrimidine derivatives, see: Světlík *et al.* (1991 ▶).
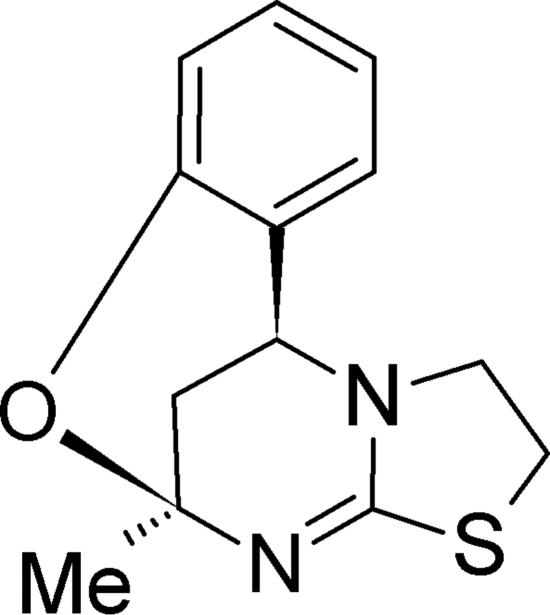


## Experimental

### 

#### Crystal data

C_13_H_14_N_2_OS
*M* *_r_* = 246.32
Monoclinic, 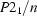

*a* = 14.307 (2) Å
*b* = 5.991 (1) Å
*c* = 15.203 (2) Å
β = 113.36 (1)°
*V* = 1196.3 (3) Å^3^
*Z* = 4
Mo *K*α radiation
μ = 0.26 mm^−1^
*T* = 296 K
0.30 × 0.25 × 0.20 mm

#### Data collection

Siemens P4 diffractometer
Absorption correction: none
4529 measured reflections
3483 independent reflections
2517 reflections with *I* > 2σ(*I*)
*R*_int_ = 0.028
3 standard reflections every 97 reflections intensity decay: none

#### Refinement

*R*[*F* ^2^ > 2σ(*F* ^2^)] = 0.053
*wR*(*F* ^2^) = 0.161
*S* = 1.05
3483 reflections
155 parameters
H-atom parameters constrained
Δρ_max_ = 0.37 e Å^−3^
Δρ_min_ = −0.24 e Å^−3^

### 

Data collection: *XSCANS* (Siemens, 1991 ▶); cell refinement: *XSCANS*; data reduction: *XSCANS*; program(s) used to solve structure: *SHELXS97* (Sheldrick, 2008 ▶); program(s) used to refine structure: *SHELXL97* (Sheldrick, 2008 ▶); molecular graphics: *PLATON* (Spek, 2009 ▶); software used to prepare material for publication: *SHELXL97*.

## Supplementary Material

Crystal structure: contains datablocks global, I. DOI: 10.1107/S1600536809044729/im2147sup1.cif

Structure factors: contains datablocks I. DOI: 10.1107/S1600536809044729/im2147Isup2.hkl

Additional supplementary materials:  crystallographic information; 3D view; checkCIF report

## Figures and Tables

**Fig. 1.**
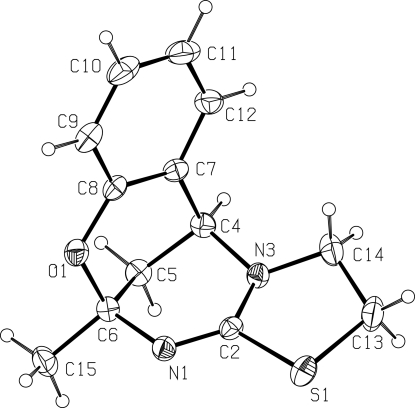
Displacement ellipsoid plot of (I) with the labelling scheme for the non-H atoms, which are drawn as 35% probability ellipsoids.

## supplementary crystallographic information

### Comment

4-Aryl-3,4-dihydropyrimidine-2(1*H*)-ones and -thiones, known as Biginelli compounds, display a wide spectrum of significant pharmacological activities (Kappe, 2000). For example, these pyrimidine derivatives were assayed as antihypertensive agents, selective α_1a_-adrenergic receptor antagonists, neuropeptide Y antagonists and were used as a lead for development of anticancer drugs (Kappe, 2000). Recently, the Biginelli products have also been found to be potent hepatitis B replication inhibitors (Deres *et al.*, 2003). As each of the above activities originates from stereo-selective binding of the drug molecule to its specific receptor, it is of interest to design a conformationally restricted probe molecule in order to examine geometric requirements of the given receptor binding site. Since we had previously synthesized such a rigid type of dihydropyrimidine, (I) (Světlík *et al.*, 1991), we decided to examine the structure of this novel heterocyclic system by an X-ray analysis.

As mentioned above, from the pharmacological point of view the most important aspect of the molecular structure (Fig.1) concerns three-dimensional disposition of the key functional (pharmacophoric) elements (hydrophobic groups and heteroatoms able to form hydrogen bonds) which in turn depends on torsional (conformational) and bonding characteristics of the molecule. First, the central heterocycle assumes an unsymmetrical half-chair conformation in which atoms C6, N1, C2 and N3 are coplanar with r.m.s. deviation of 0.005 (1) Å, while atoms C4 and C5 are displaced from this plane by -0.281 (3) and 0.520 (3) Å, respectively. Next, the five-membered thiazolo ring adopts a flat-envelope conformation with atom C14 deviating by 0.423 (3) Å from the mean plane of the remaining four atoms [r.m.s. deviation 0.004 (1) Å]. Finally, the phenyl ring on C4 is, due to its pseudoaxial position and the O-atom bridge (Fig.1), fixed approximately in a perpendicular orientation with respect to the mean plane of the dihydropyrimidine ring [dihedral angle 84.6 (1)°]. All the above conformations arise from the rigidity of the polycyclic system as well as the bonding pattern within the π-electron portion of the fused heterocyclic substructure. Thus, the N1=C2 bond length of 1.287 (2) Å corresponds to pure double bond (Burke-Laing & Laing, 1976), while the formally single bonds S1—C2 and C2—N3 have partial double-bond character (Abrahams, 1956; Burke-Laing & Laing, 1976), obviously due to partial *sp*^2^-hybridization state of N3 and hence some degree of conjugation between N3 and S1.

As there is no classical hydrogen-bond donor site in the molecule, the crystal packing is governed by weak C–H···O and C–H···N contacts and van der Waals interactions.

### Experimental

Synthesis of the title compound, (I), has been described (Světlík *et al.*, 1991). In short, a solution of methyl 3,4,5,6-tetrahydro-2-methyl-2,6-methano-4-thioxo- 2*H*-1,3,5-benzoxadiazocine-11-carboxylate (1.0 g, 3.59 mmol) and 1,2-dibromoethane (0.35 ml, 4.0 mmol) in dimethylformamide was refluxed for 40 minutes. The resulting hydrobromide was treated with aqueous sodium carbonate to furnish the corresponding free base (42% yield; m.p. 513–514 K). Single crystals suitable for an X-ray analysis were obtained by recrystallization from acetonitrile.

### Refinement

H atoms were visible in difference maps and were subsequently treated as riding atoms with distances C—H = 0.93 Å (CH_arom_), 0.97 (CH_2_) or 0.98 Å (CH) and 0.96 Å (CH_3_); *U*_iso_ of the H atoms were set to 1.2 (1.5 for the methyl H atoms) times *U*_eq_ of the parent atom.

### Crystal data

**Table:**

C_13_H_14_N_2_OS	*F*(000) = 520
*M**_r_* = 246.32	*D*_x_ = 1.368 Mg m^−^^3^
Monoclinic, *P*2_1_/*n*	Melting point: 513 K
Hall symbol: -P 2yn	Mo *K*α radiation, λ = 0.71073 Å
*a* = 14.307 (2) Å	Cell parameters from 20 reflections
*b* = 5.991 (1) Å	θ = 7–18°
*c* = 15.203 (2) Å	µ = 0.26 mm^−^^1^
β = 113.36 (1)°	*T* = 296 K
*V* = 1196.3 (3) Å^3^	Prism, colourless
*Z* = 4	0.30 × 0.25 × 0.20 mm

### Data collection

**Table:**

Siemens P4 diffractometer	*R*_int_ = 0.028
Radiation source: fine-focus sealed tube	θ_max_ = 30.0°, θ_min_ = 1.7°
graphite	*h* = −1→20
ω/2θ scans	*k* = −1→8
4529 measured reflections	*l* = −21→20
3483 independent reflections	3 standard reflections every 97 reflections
2517 reflections with *I* > 2σ(*I*)	intensity decay: none

### Refinement

**Table:**

Refinement on *F*^2^	Primary atom site location: structure-invariant direct methods
Least-squares matrix: full	Secondary atom site location: difference Fourier map
*R*[*F*^2^ > 2σ(*F*^2^)] = 0.053	Hydrogen site location: inferred from neighbouring sites
*wR*(*F*^2^) = 0.161	H-atom parameters constrained
*S* = 1.05	*w* = 1/[σ^2^(*F*_o_^2^) + (0.0855*P*)^2^ + 0.232*P*] where *P* = (*F*_o_^2^ + 2*F*_c_^2^)/3
3483 reflections	(Δ/σ)_max_ = 0.001
155 parameters	Δρ_max_ = 0.37 e Å^−^^3^
0 restraints	Δρ_min_ = −0.23 e Å^−^^3^

### Special details

**Table:**

Geometry. All e.s.d.'s (except the e.s.d. in the dihedral angle between two l.s. planes) are estimated using the full covariance matrix. The cell e.s.d.'s are taken into account individually in the estimation of e.s.d.'s in distances, angles and torsion angles; correlations between e.s.d.'s in cell parameters are only used when they are defined by crystal symmetry. An approximate (isotropic) treatment of cell e.s.d.'s is used for estimating e.s.d.'s involving l.s. planes.
Refinement. Refinement of *F*^2^ against ALL reflections. The weighted *R*-factor *wR* and goodness of fit *S* are based on *F*^2^, conventional *R*-factors *R* are based on *F*, with *F* set to zero for negative *F*^2^. The threshold expression of *F*^2^ > σ(*F*^2^) is used only for calculating *R*-factors(gt) *etc*. and is not relevant to the choice of reflections for refinement. *R*-factors based on *F*^2^ are statistically about twice as large as those based on *F*, and *R*- factors based on ALL data will be even larger.

### Fractional atomic coordinates and isotropic or equivalent isotropic displacement parameters (Å^2^)

**Table:**

	*x*	*y*	*z*	*U*_iso_*/*U*_eq_	
N1	0.24822 (12)	0.3305 (3)	−0.01176 (11)	0.0354 (3)	
C2	0.20695 (13)	0.4743 (3)	0.02460 (12)	0.0316 (4)	
N3	0.18468 (12)	0.6936 (3)	0.00041 (10)	0.0345 (3)	
C4	0.18874 (14)	0.7633 (3)	−0.09082 (13)	0.0337 (4)	
H4	0.1918	0.9264	−0.0938	0.040*	
C5	0.28506 (14)	0.6602 (3)	−0.09323 (14)	0.0370 (4)	
H5A	0.3443	0.7084	−0.0381	0.044*	
H5B	0.2936	0.7054	−0.1509	0.044*	
C6	0.27290 (14)	0.4073 (3)	−0.09162 (13)	0.0338 (4)	
C7	0.09818 (14)	0.6755 (3)	−0.17604 (12)	0.0336 (4)	
C8	0.10420 (13)	0.4666 (3)	−0.21460 (12)	0.0337 (4)	
O1	0.19045 (11)	0.3365 (2)	−0.18067 (9)	0.0393 (3)	
C9	0.02109 (17)	0.3840 (4)	−0.29390 (14)	0.0450 (5)	
H9	0.0249	0.2451	−0.3195	0.054*	
C10	−0.06598 (17)	0.5104 (5)	−0.33332 (15)	0.0552 (6)	
H10	−0.1210	0.4555	−0.3857	0.066*	
C11	−0.07307 (17)	0.7163 (5)	−0.29657 (16)	0.0565 (6)	
H11	−0.1324	0.8000	−0.3241	0.068*	
C12	0.00859 (16)	0.7986 (4)	−0.21826 (15)	0.0447 (5)	
H12	0.0036	0.9379	−0.1935	0.054*	
S1	0.17224 (4)	0.40002 (11)	0.12054 (4)	0.04879 (19)	
C13	0.1245 (2)	0.6777 (5)	0.12630 (16)	0.0572 (6)	
H13A	0.0601	0.6694	0.1333	0.069*	
H13B	0.1727	0.7598	0.1802	0.069*	
C14	0.11074 (18)	0.7907 (4)	0.03283 (16)	0.0497 (5)	
H14A	0.0422	0.7665	−0.0145	0.060*	
H14B	0.1220	0.9501	0.0425	0.060*	
C15	0.36653 (17)	0.2829 (4)	−0.08909 (17)	0.0490 (5)	
H15A	0.4235	0.3180	−0.0308	0.074*	
H15B	0.3813	0.3269	−0.1429	0.074*	
H15C	0.3539	0.1251	−0.0919	0.074*	

### Atomic displacement parameters (Å^2^)

**Table:**

	*U*^11^	*U*^22^	*U*^33^	*U*^12^	*U*^13^	*U*^23^
N1	0.0407 (8)	0.0327 (8)	0.0351 (7)	0.0028 (6)	0.0173 (6)	0.0016 (6)
C2	0.0295 (8)	0.0363 (9)	0.0270 (7)	−0.0032 (7)	0.0089 (6)	−0.0013 (6)
N3	0.0366 (8)	0.0346 (8)	0.0323 (7)	0.0010 (6)	0.0137 (6)	−0.0053 (6)
C4	0.0369 (9)	0.0261 (8)	0.0378 (9)	−0.0025 (7)	0.0144 (7)	−0.0001 (7)
C5	0.0347 (9)	0.0377 (10)	0.0399 (9)	−0.0056 (7)	0.0161 (7)	0.0001 (7)
C6	0.0347 (8)	0.0339 (9)	0.0346 (8)	0.0010 (7)	0.0156 (7)	−0.0015 (7)
C7	0.0361 (9)	0.0342 (9)	0.0301 (8)	−0.0007 (7)	0.0127 (7)	0.0052 (7)
C8	0.0368 (9)	0.0386 (9)	0.0273 (7)	−0.0058 (7)	0.0145 (7)	0.0006 (7)
O1	0.0456 (7)	0.0358 (7)	0.0353 (6)	−0.0002 (6)	0.0148 (6)	−0.0066 (5)
C9	0.0527 (12)	0.0514 (12)	0.0324 (9)	−0.0127 (10)	0.0185 (8)	−0.0035 (8)
C10	0.0420 (11)	0.0852 (18)	0.0314 (9)	−0.0130 (12)	0.0073 (8)	0.0010 (10)
C11	0.0391 (11)	0.0803 (18)	0.0417 (11)	0.0066 (11)	0.0072 (9)	0.0170 (12)
C12	0.0451 (10)	0.0470 (11)	0.0416 (10)	0.0078 (9)	0.0168 (8)	0.0104 (9)
S1	0.0577 (3)	0.0566 (4)	0.0390 (3)	−0.0027 (3)	0.0265 (2)	0.0012 (2)
C13	0.0631 (14)	0.0717 (16)	0.0452 (11)	0.0011 (12)	0.0306 (11)	−0.0175 (11)
C14	0.0528 (12)	0.0471 (12)	0.0556 (12)	0.0061 (10)	0.0283 (10)	−0.0107 (10)
C15	0.0442 (11)	0.0516 (13)	0.0592 (13)	0.0102 (10)	0.0289 (10)	0.0000 (10)

### Geometric parameters (Å, °)

**Table:**

N1—C2	1.287 (2)	C8—C9	1.405 (3)
N1—C6	1.466 (2)	C9—C10	1.375 (3)
C2—N3	1.368 (2)	C9—H9	0.9300
C2—S1	1.7738 (18)	C10—C11	1.375 (4)
N3—C14	1.454 (2)	C10—H10	0.9300
N3—C4	1.471 (2)	C11—C12	1.387 (3)
C4—C7	1.518 (2)	C11—H11	0.9300
C4—C5	1.524 (3)	C12—H12	0.9300
C4—H4	0.9800	S1—C13	1.814 (3)
C5—C6	1.527 (3)	C13—C14	1.515 (3)
C5—H5A	0.9700	C13—H13A	0.9700
C5—H5B	0.9700	C13—H13B	0.9700
C6—O1	1.462 (2)	C14—H14A	0.9700
C6—C15	1.520 (3)	C14—H14B	0.9700
C7—C12	1.395 (3)	C15—H15A	0.9600
C7—C8	1.399 (3)	C15—H15B	0.9600
C8—O1	1.375 (2)	C15—H15C	0.9600
			
C2—N1—C6	116.60 (16)	C10—C9—C8	119.5 (2)
N1—C2—N3	128.72 (17)	C10—C9—H9	120.3
N1—C2—S1	120.73 (15)	C8—C9—H9	120.3
N3—C2—S1	110.54 (13)	C11—C10—C9	121.1 (2)
C2—N3—C14	114.61 (17)	C11—C10—H10	119.4
C2—N3—C4	115.73 (14)	C9—C10—H10	119.4
C14—N3—C4	120.80 (16)	C10—C11—C12	119.7 (2)
N3—C4—C7	111.52 (14)	C10—C11—H11	120.2
N3—C4—C5	106.36 (14)	C12—C11—H11	120.2
C7—C4—C5	108.24 (15)	C11—C12—C7	121.0 (2)
N3—C4—H4	110.2	C11—C12—H12	119.5
C7—C4—H4	110.2	C7—C12—H12	119.5
C5—C4—H4	110.2	C2—S1—C13	92.48 (10)
C4—C5—C6	106.99 (15)	C14—C13—S1	106.02 (14)
C4—C5—H5A	110.3	C14—C13—H13A	110.5
C6—C5—H5A	110.3	S1—C13—H13A	110.5
C4—C5—H5B	110.3	C14—C13—H13B	110.5
C6—C5—H5B	110.3	S1—C13—H13B	110.5
H5A—C5—H5B	108.6	H13A—C13—H13B	108.7
O1—C6—N1	107.76 (14)	N3—C14—C13	107.34 (18)
O1—C6—C15	105.11 (15)	N3—C14—H14A	110.2
N1—C6—C15	108.93 (16)	C13—C14—H14A	110.2
O1—C6—C5	109.15 (15)	N3—C14—H14B	110.2
N1—C6—C5	113.02 (15)	C13—C14—H14B	110.2
C15—C6—C5	112.47 (17)	H14A—C14—H14B	108.5
C12—C7—C8	118.55 (18)	C6—C15—H15A	109.5
C12—C7—C4	121.83 (18)	C6—C15—H15B	109.5
C8—C7—C4	119.62 (16)	H15A—C15—H15B	109.5
O1—C8—C7	123.01 (16)	C6—C15—H15C	109.5
O1—C8—C9	116.78 (18)	H15A—C15—H15C	109.5
C7—C8—C9	120.17 (18)	H15B—C15—H15C	109.5
C8—O1—C6	117.24 (14)		
			
C6—N1—C2—N3	−1.7 (3)	C12—C7—C8—O1	177.30 (17)
C6—N1—C2—S1	179.22 (12)	C4—C7—C8—O1	−2.0 (3)
N1—C2—N3—C14	161.48 (18)	C12—C7—C8—C9	−0.3 (3)
S1—C2—N3—C14	−19.39 (19)	C4—C7—C8—C9	−179.59 (17)
N1—C2—N3—C4	13.7 (3)	C7—C8—O1—C6	9.4 (2)
S1—C2—N3—C4	−167.15 (12)	C9—C8—O1—C6	−172.92 (16)
C2—N3—C4—C7	73.81 (19)	N1—C6—O1—C8	81.42 (18)
C14—N3—C4—C7	−71.8 (2)	C15—C6—O1—C8	−162.51 (16)
C2—N3—C4—C5	−44.00 (19)	C5—C6—O1—C8	−41.7 (2)
C14—N3—C4—C5	170.38 (16)	O1—C8—C9—C10	−177.58 (18)
N3—C4—C5—C6	62.31 (18)	C7—C8—C9—C10	0.1 (3)
C7—C4—C5—C6	−57.65 (19)	C8—C9—C10—C11	0.1 (3)
C2—N1—C6—O1	−97.83 (18)	C9—C10—C11—C12	−0.2 (4)
C2—N1—C6—C15	148.63 (17)	C10—C11—C12—C7	0.1 (3)
C2—N1—C6—C5	22.9 (2)	C8—C7—C12—C11	0.2 (3)
C4—C5—C6—O1	66.42 (18)	C4—C7—C12—C11	179.48 (19)
C4—C5—C6—N1	−53.5 (2)	N1—C2—S1—C13	179.86 (16)
C4—C5—C6—C15	−177.33 (16)	N3—C2—S1—C13	0.65 (14)
N3—C4—C7—C12	91.3 (2)	C2—S1—C13—C14	16.47 (17)
C5—C4—C7—C12	−152.05 (17)	C2—N3—C14—C13	32.1 (2)
N3—C4—C7—C8	−89.41 (19)	C4—N3—C14—C13	178.03 (17)
C5—C4—C7—C8	27.3 (2)	S1—C13—C14—N3	−28.8 (2)
